# Peripheral denervation participates in heterotopic ossification in a spinal cord injury model

**DOI:** 10.1371/journal.pone.0182454

**Published:** 2017-08-30

**Authors:** Charlotte Debaud, Marjorie Salga, Laurent Begot, Xavier Holy, Malha Chedik, Nicolas de l’Escalopier, Fréderic Torossian, Jean-Pierre Levesque, Jean-Jacques Lataillade, Marie-Caroline Le Bousse-Kerdilès, François Genêt

**Affiliations:** 1 Spine Division Orthopaedic Surgery Department, Hôpital Européen Georges Pompidou, APHP, Paris, France; 2 University of Versailles Saint Quentin en Yvelines, U1179 INSERM, UFR des Sciences de la Santé – Simone Veil, Montigny-le-Bretonneux, France; 3 Rehabilitation Service, Hôpital Raymond Poincaré, APHP, CIC-IT 1429, Garches, France; 4 Institut de Recherche Biomédicale des Armées, Brétigny-sur-Orge, France; 5 Orthopaedic Surgery Department, Hôpital d’Instruction des Armées PERCY, Clamart, France; 6 University of Paris-Sud, INSERM UMR-S/MD 1197, Hôpital Paul Brousse, APHP, Villejuif, France; 7 Blood and Bone Diseases Program, Mater Research Institute, University of Queensland, Woolloongabba and School of Medicine, University of Queensland, Herston, Queensland, Australia; 8 University of Paris-Sud, Unité mixte Inserm/SSA 1197, IRBA/CTSA/HIA Percy, École du Val de Grâce, Clamart, France; University of Minnesota Medical Center, UNITED STATES

## Abstract

We previously reported the development of a new acquired neurogenic HO (NHO) mouse model, combining spinal cord transection (SCI) and chemical muscle injury. Pathological mechanisms responsible for ectopic osteogenesis after central neurological damage are still to be elucidated. In this study, we first hypothesized that peripheral nervous system (PNS) might convey pathological signals from injured spinal cord to muscles in NHO mouse model. Secondly, we sought to determine whether SCI could lead to intramuscular modifications of BMP2 signaling pathways. Twenty one C57Bl6 mice were included in this protocol. Bilateral cardiotoxin (CTX) injection in hamstring muscles was associated with a two-stage surgical procedure, combining thoracic SCI with unilateral peripheral denervation. Volumes of HO (Bone Volume, BV) were measured 28 days after surgery using micro-computed tomography imaging techniques and histological analyses were made to confirm intramuscular osteogenesis. Volume comparisons were conducted between right and left hind limb of each animal, using a Wilcoxon signed rank test. Quantitative polymerase chain reaction (qPCR) was performed to explore intra muscular expression of BMP2, Alk3 and Id1. Nineteen mice survive the complete SCI and peripheral denervation procedure. When CTX injections were done right after surgery (n = 7), bilateral HO were detected in all animals after 28 days. Micro-CT measurements showed significantly increased BV in denervated paws (1.47 mm3 +/- 0.5) compared to contralateral sides (0.56 mm3 +/-0.4), p = 0.03. When peripheral denervation and CTX injections were performed after sham SCI surgery (n = 6), bilateral HO were present in three mice at day 28. Quantitative PCR analyses showed no changes in intra muscular BMP2 expression after SCI as compared to control mice (shamSCI). Peripheral denervation can be reliably added to spinal cord transection in NHO mouse model. This new experimental design confirms that neuro inflammatory mechanisms induced by central or peripheral nervous system injury plays a key role in triggering ectopic osteogenesis.

## Introduction

Neurogenic heterotopic ossification (NHO) can be defined as the ectopic formation of lamellar bone in non-osseous tissues following traumatic brain or medullar injury. It is a debilitating and costly condition, which has been reported to concern nearly 10% to 40% of neurologically impaired patients [1–4]. Preferentially located in extracapsular tissues around hip and elbow [5], NHO can cause articular ankyloses or vascular and nervous compressions [6]. Pathophysiological mechanisms involved in this process remain elusive which makes it difficult to elaborate efficient preventive strategies [7, 8]. Therefore, surgical excision is usually performed to control symptomatic NHO, with a significant risk of perioperative complications and potential relapses even late after surgery [5, 9–12]. The persistent lack of non-invasive treatment for neurologically damaged and fragile patients has driven efforts to develop experimental studies on HO animal models [13, 14].

In that specific research field, last decades have been marked by the identification of Bone Morphogenetic Proteins (BMPs), which turned out to be important molecular actors of osteogenesis regulation. This group of proteins belongs to the Transforming Growth Factor-β (TGF-β) superfamily and first observations of their osteo-inductive effects were published in the mid-1960s by Urist [15]. As rapid progress were made towards understanding BMP molecular signalling pathways [16], HO animal models involving BMP overexpression have been developed, either through matrigel heterotopic implantation or transduced cells [17–21]. Yet, it seems difficult to transpose those experimental models to clinical applications since they don’t reflect the complexity of regulation pathways involved in neurologically injured patients.

For that reason, we recently developed an acquired NHO mouse model [22] featuring central neurologic and peripheral limb injuries as observed in patients who suffer high-speed accident. We demonstrated that intramuscular osteogenesis could be reliably induced in C57Bl6 mice by combining thoracic spinal cord transection with intramuscular injection of cardiotoxin (CTX). CTX is a potent polypeptidic snake-venom which causes muscle damage and localized inflammation. Besides macrophage involvement, our previous works underlined the critical role of central nervous tissue damage in triggering the shift from muscular regeneration towards osteogenic process [22].

Several hypotheses have been discussed about molecular signals conveyed from injured spinal cord to musculoskeletal effectors. Gautschi et al. supported humoral regulation of ectopic osteogenesis, highlighting osteo-inductive effect of the cerebrospinal fluid (CSF) from patients with severe TBI [23, 24]. Disruption of blood brain barrier following central nervous system injury could lead to the systemic release of osteogenic factors but also to the cellular activation of circulating progenitors. More recent works by Kan et al. have shown that concentration of a neuromodulator called substance P was significantly higher in blood from NHO patients and similarly in different HO mouse models [25]. As substance P is known to be involved in bone remodelling regulation [26], it seems logical to suppose that it plays a key role in neurogenic HO formation. Indeed, Salisbury et al. demonstrated that substance P could be released by activated sensory neurons upon BMP stimulation [27] and promote intra muscular NHO formation. Nevertheless, the role of sensory neurons in the context of spinal cord injury is not as clear. Below the level of injury, afferent inputs are released from cortical control and conveyed dysregulated signals to muscular effectors, whom involvement in NHO formation remains to be elucidated.

Another plausible hypothesis regarding NHO pathophysiology is an induced dysregulation of BMP signalling pathway, which is supported by numerous experimental works on hereditary HO animal models [16, 28–30], as well as recent identification of ACVR1 mutation responsible for fibrodysplasia ossificans progressiva [31–33]. In patients affected by this disease, pathologic upregulation of BMP-4 signal leads to multiple heterotopic endochondral ossifications. However, no evidence has yet been published regarding abnormal BMP expression in acquired neurogenic HO.

Considering these data, we thought relevant to focus on both PNS regulation and BMP expression in our NHO mouse model. To evaluate the influence of peripheral deafferentation below the level of central injury, we first sought to develop a new surgical procedure of mouse hind limb peripheral denervation, which could be combined with spinal cord transection and muscular cardiotoxin injection. To evaluate the influence of central neurological damage on BMP signalling pathway in muscles, we measured BMP2 and Alk3 (activin-like kinase 3, type 1 BMP receptor) muscular expression rates, in early stage following spinal cord injury or sham surgery.

## Materials and methods

### Study groups

To evaluate the role of peripheral innervation on ectopic bone volume after spinal cord injury (SCI) we randomly assigned 21 females C57Bl6 mice of 5 weeks-old to three groups. Experimental design in group 1 (n = 7) included spinal cord transection (SCI surgery), right hind-limb peripheral denervation and bilateral hamstrings cardiotoxin injections (CTX, C9759, Sigma-Aldrich). Same surgical procedures were performed in group 2 (n = 7), except that bilateral CTX injections were realized 10 days after surgery. In group 3 (n = 7), we performed control procedure with SHAM SCI surgery, right hind-limb peripheral denervation and bilateral hamstrings CTX injections.

To evaluate whether spinal cord injury could induce specific dysregulation of intramuscular BMP2 signaling pathway in wild type mice, we quantified BMP2, ALK3 and Id1 RNA expression in muscle samples of 20 C57Bl6 females eighteen hours after SCI (n = 10) comparatively to SHAM SCI surgery (n = 10). We choose this early-stage time point considering that pathophysiological changes resulting from the central nervous system injury settle quickly in the acute post traumatic phase.

### Surgical procedures

**Fig 1:**
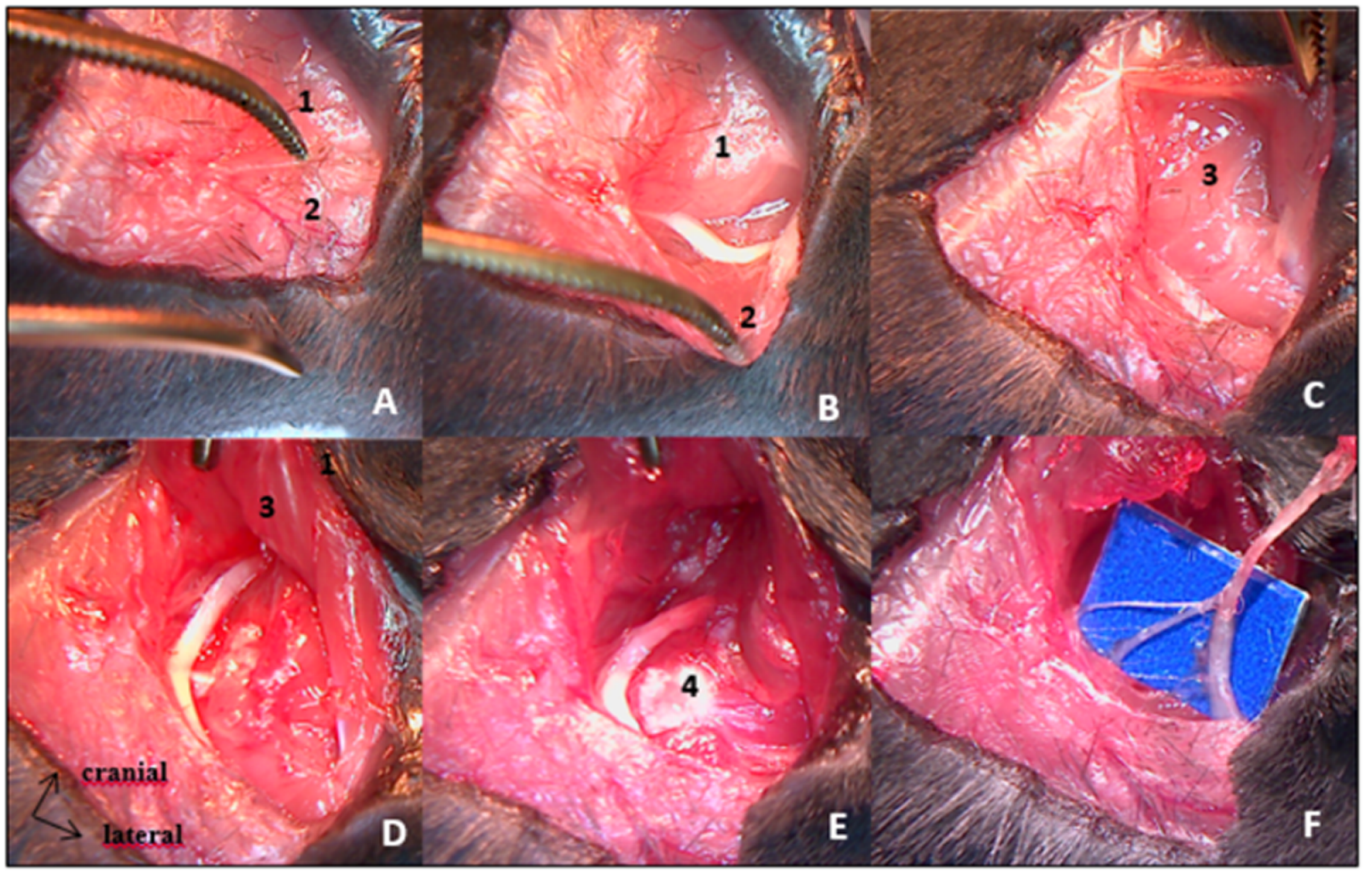
Sciatic nerve excision procedure by posterior approach. A: skin incision over posterior surface of proximal hind limb exposes superficial muscular layers [gluteus maximus (1) and biceps femoralis (2)]. B: Biceps femoralis retraction allows sciatic nerve identification. C: Pelvic insertion of gluteus maximus muscle is freed to expose deep muscular layers [gluteus medius and gluteus minimus (3)]. D & E: Greater trochanter (4) exposure after deep muscles retraction. Sciatic nerve is followed throughout its course towards the sciatic notch. E: Proximal cut of sciatic nerve. F: Divisional branches of the sciatic nerve to hamstring muscles.

**Fig 2:**
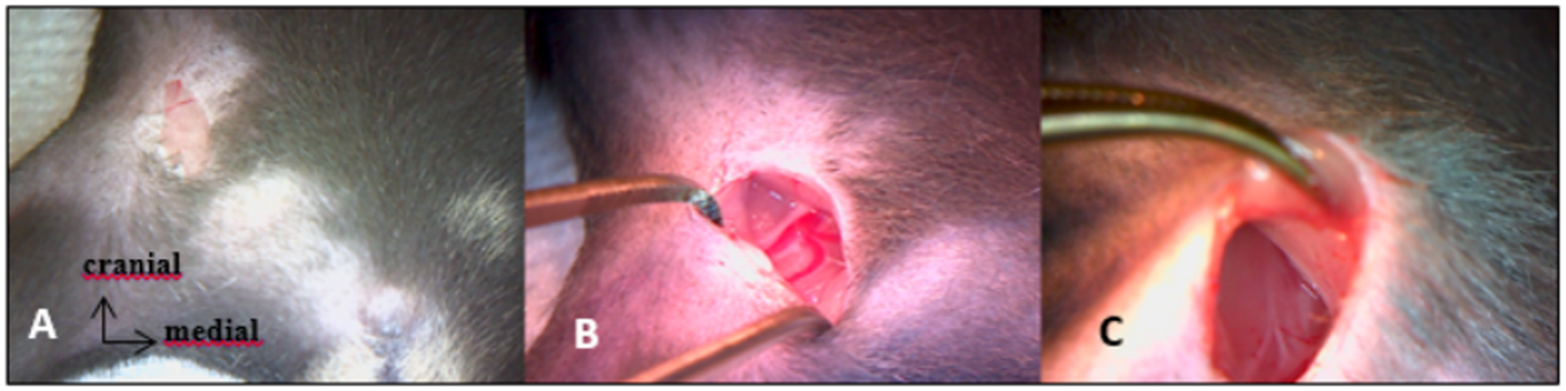
Anterior approach of femoral nerve. A: skin incision through inguinal fold. B: femoral neuro-vascular bundle exposure. C: proximal part of femoral nerve laying over psoas muscle.

All procedures were realized under the microscope. Mice were anesthetized with isoflurane gas (2.5% with oxygen 21% balanced), on hot plate to maintain body temperature around 37°C during surgery. Skin incision was made on top of thoracic kyphosis and spinal cord was exposed by removing inter-lamar ligament in between posterior laminae of two contiguous vertebrae. Complete transection of spinal cord was achieved using surgical blade. Hemostasis was obtained using cautious compression with cotton plug. SHAM SCI surgery consisted in posterior approach of vertebral column ending before inter-lamar ligament removal, without spinal cord transection. We pursued the procedure with right hind-limb complete denervation, beginning with sciatic nerve excision (Fig 1). After right tight skin incision, sciatic nerve was exposed extensively, followed from mid tight to gluteal fossa. All divisional branches were identified and transected. Large removal of sciatic nerve was then performed, after realizing proximal transection as far upstream as possible, in contact with the iliac wing. Second step of this procedure was femoral nerve excision performed through an anterior inguinal approach, extended into the retroperitoneal space (Fig 2). Femoral nerve was exposed on the psoas muscle and removed from there to the iliac vessels. Muscles and skin were sutured at the end of each step.

### Injections

We reconstituted a solution of Cardiotoxin from *naja mossambica* (CTX, C9759, Sigma-Aldrich) at 10μmol/L. 75μL of this solution was injected in both hamstring muscles. For groups 1 and 3, injections were performed during surgery, as hamstring muscles were exposed for sciatic nerve transection procedure. For group 2, CTX were performed 10 days after surgery, percutaneously. Antibiotics prophylaxis was achieved through intraperitoneal injection of ciprofloxacin (Ciflox^®^; 10mg/kg)

### Postoperative treatment

Animals were kept in standard plastic cages, under controlled environmental conditions with food and water ad libitum. As animals from groups 1 and 2 were paraplegic, we practiced manual bladder emptying twice a day until sacrifices. To prevent urinary tract infection, sulfamethoxazole/trimethoprim (Bactrim^®^; 50/10 mg/kg) was added in drinking water.

Surveillance score was used daily to ensure the absence of motor recuperation, wound infections, weight over loss or suffering signs. Mice from three groups were euthanized 28 days after surgery. This procedure was performed under anesthesia, first using isoflurane 2.5% mixed with oxygen 100%. After exposition of intrathoracic organs, isoflurane inhalation was relayed by urethane intra-cardiac injection (200mg/mL) and exsanguination was then performed. All these steps were conducted within a chemical fume hood utilizing sash, by competent staff wearing protective equipment. Syringes used for urethane injections were safety engineered. All Precautions were taken in order to keep exsanguinated animals out of sight or smell from others mice. Animals' hindquarters were harvested, immersed 24h in paraformaldehyde 4% and conserved in phosphate-buffered saline.

### Micro-tomography

Micro-tomography was performed on each hindquarter with a skyscan 1174 X-ray computed microtomograph (Bruker MicroCT, Kontich, Belgium). Scanning parameters were 50kV, 800μA and a 0.5mm aluminum filter. Measurement calibration was based on bone mineral density of hydroxyapatite phantoms (Bruker-Micro CT BMD calibration phantoms, concentrations of CaHA: 0.25 and 0.75 g.cm-3). To distinguish mineralized from soft tissues, we applied segmentation with threshold grey level density values set up at 45 and 220. The CTAn Software was used to measure the volume of HO (Bone Volume, BV mm3) developed in both hamstrings of each mouse. To distinguish HO from soft tissues, we followed CTAn software processing steps: regions of interest were manually delineated on each microCT transversal slice, around heterotopic mineralization, taking care not to include lower limb bones, easily identified. Binarisation were applied on the volume of interest delineated to separate HO from background. Threshold grey level density values, set up at 45 and 220, were defined after software calibration by means of hydroxyapatite phantoms (0.25 g/cm3 and 0.75 g/cm3).

### Histological analyses

Whole hind limbs were embedded in paraffin after EDTA 5% decalcification for about ten days (Excelsior Shandon robot), sectioned at 5 μm (LEICA 2025 microtome), stained with Hematoxylin-Eosin-Saffron, and observed under an optical microscope.

### Quantitative polymerase chain reaction

To isolate total ribonucleic acid (RNA), hamstring muscles were removed from animals immediately after scarification, and homogenized in 500 μL of Trizol reagent (Life Technologies; Grand Island, New York) according to the manufacturer’s instructions. Isolated RNA was quantified and normalized to synthesize complementary deoxyribonucleic acid, using RNeasy mini kit (Qiagen^®^). Real-time polymerase chain reaction (RT-PCR) was performed to quantify the expression of BMP2, ALK3 and ID1 genes in muscle samples using a SYBR Green Supermix iTaq Universal (BioRad^®^). Gene expression was normalized to glyceraldehyde 3-phosphate dehydrogenase and 18S, housekeeping genes. For each tested gene, primers sequences and efficiencies (E) are presented in Table 1.

**Table 1:** Primers sequences and efficiencies used for quantitative RT-PCR.

Gene	Forward primer	Reverse primer	E
BMP2	5’-CATCACGAAGAAGCCGTGGA-3’	3’-TGAGAAACTCGTCACTGGGG-5’	1,96
ID1	5’-GGTGGTACTTGGTCTGTCGG-3’	3’-CCTTGCTCACTTTGCGGTTC-5’	1,88
AlK3	5’-TGAGACAGCAGGACCAGTCA-3’	3’-GATTCTGCCCTTGAACATGAGA-5’	1,92
18S	5’-CATTCGAACGTCTGCCCTATC-3’	3’-CTCCCTCTCCGGAATCGAAC-5’	1,99
GAPDH	5’-TGACGTGCCGCCTGGAGAAA-3’	3’-AGTGTAGCCCAAGATGCCCTTCAG-5’	1,99

### Statistical analyses

All calculations were performed using GraphPad Prism 6.01 software. Wilcoxon matched-pairs signed rank test was used to compare HO BV developed in innervated and denervated leg of each mouse. Statistical significance was considered when p<0.05.

### Ethics committee approval

This study protocol was reviewed and approved by the local ethics committee (University of Versailles-Saint Quentin en Yvelines) and conducted in accordance with international ethics standards for animal experimentation, number 201609231059233.

## Results

**Fig 3:**
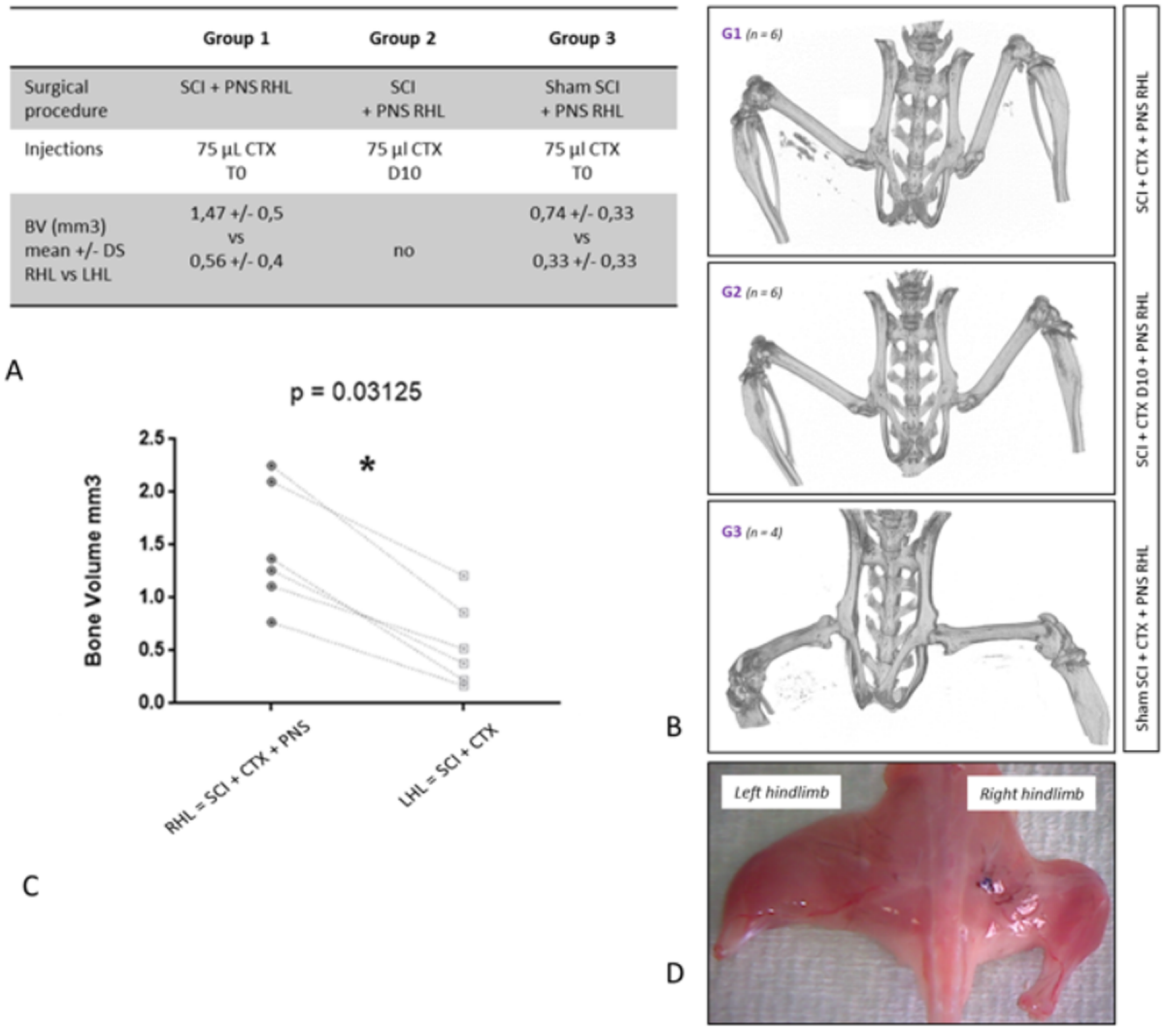
Experimental design and bone volume measurements. **Fig 3A**. SCI: spinal cord injury; PNS: peripheral nervous system; CTX: cardiotoxin. T0: CTX was injected during surgery. D10: CTX was injected 10 days after surgery BV: HO Bone Volume measured with CT scan in right hind limb (RHL) versus left hind limb (LHL). **Fig 3B**. CT scan of harvested hind limbs showed bilateral intramuscular HO in one mouse from group 1 and 3 and no HO in mouse from group 2. **Fig 3C**. HO volumes in hind limbs of 6 mice from group 1 showing significant increased BV after peripheral denervation. **Fig 3D**. Amyotrophic right hind limb after peripheral denervation 28 days after surgery.

Surgeries were performed on 21 C57Bl6 mice. 2 mice died in the early postoperative period (1 in group 1 and 1 in group 3). We suspect several factors to be involved in those premature deaths such as thermoregulation dysfunction (secondary to central nervous system injury) and surgical bleeding (epidural, medullary and gluteal vessels). It is important to note that careful dissection must be conducted during sciatic nerve exposure in the gluteal fossa. Indeed, gluteal artery is at risk of injury during this part of the surgical procedure and hemostasis gesture is difficult to achieve in this particular location. When CTX was injected 10 days after surgery (group2, n = 7), none of the samples presented intramuscular HO on micro-tomography images. In group 1 when CTX injection was done right after SCI and peripheral denervation procedures (group1, n = 6), we observed a significant increase of HO volumes in denervated right hamstrings compared to innervated left hamstrings (mean BV respectively 1.47 mm3 +/- 0.5 vs 0.56 mm3 +/- 0.4 p = 0.03; Fig 3A, 3B and 3C; S1 Table). Quantitative analysis showed that peripheral denervation, combined with SCI and CTX injection, increased heterotopic bone volume by a factor of 3.4. In group 3, when shamSCI surgery was combined with peripheral denervation and CTX injection (group 3, n = 6), all mice exhibit postoperative right hind limb complete paralysis. At the time of sacrifice (day 28), we constantly noticed a major and global muscular amyotrophy in the right hind limb compared to the contralateral side. Concerning outcome measures, micro-CT analysis revealed bilateral HOs in 3 mice out of six with the same tendency to increased BV in denervated limb compared to non-paralyzed control paws (0.74 mm^3^ +/- 0.33 vs 0.33 mm^3^+/- 0.33; not statistically significant; S1 Table).

**Fig 4:**
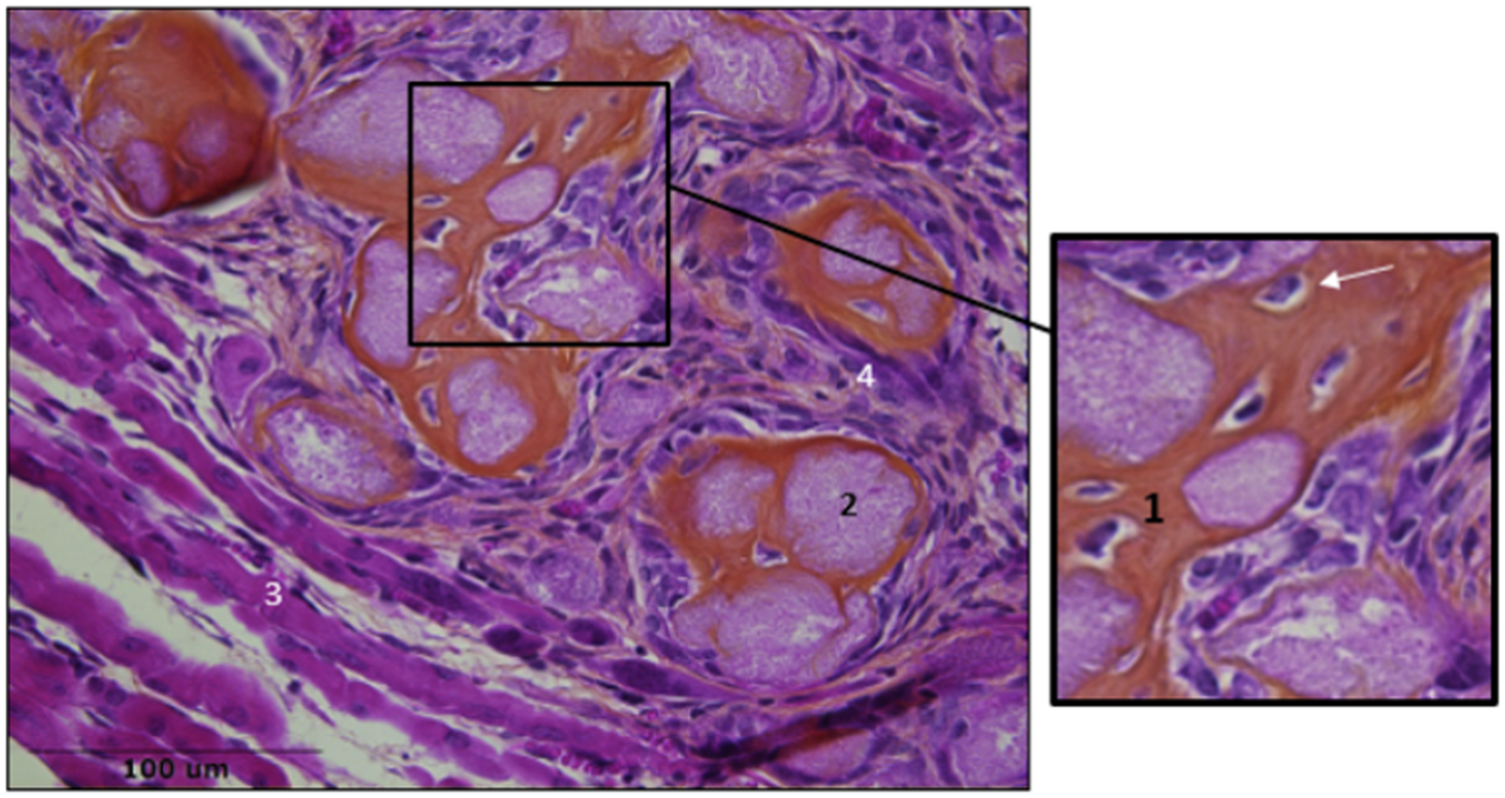
Muscular samples after SCI and CTX injection. HES staining showed bone matrix (1), osteocytes (white arrow) mineralized nodules (2), regenerated muscular fibers (3) infiltration of inflammatory cells (4).

Histologic examination at day 28 confirmed the presence of ectopic osteogenesis in muscular samples identified as HO positive by micro-CT (Fig 4) with no qualitative differences between right and left hind-limbs.

**Fig 5:**
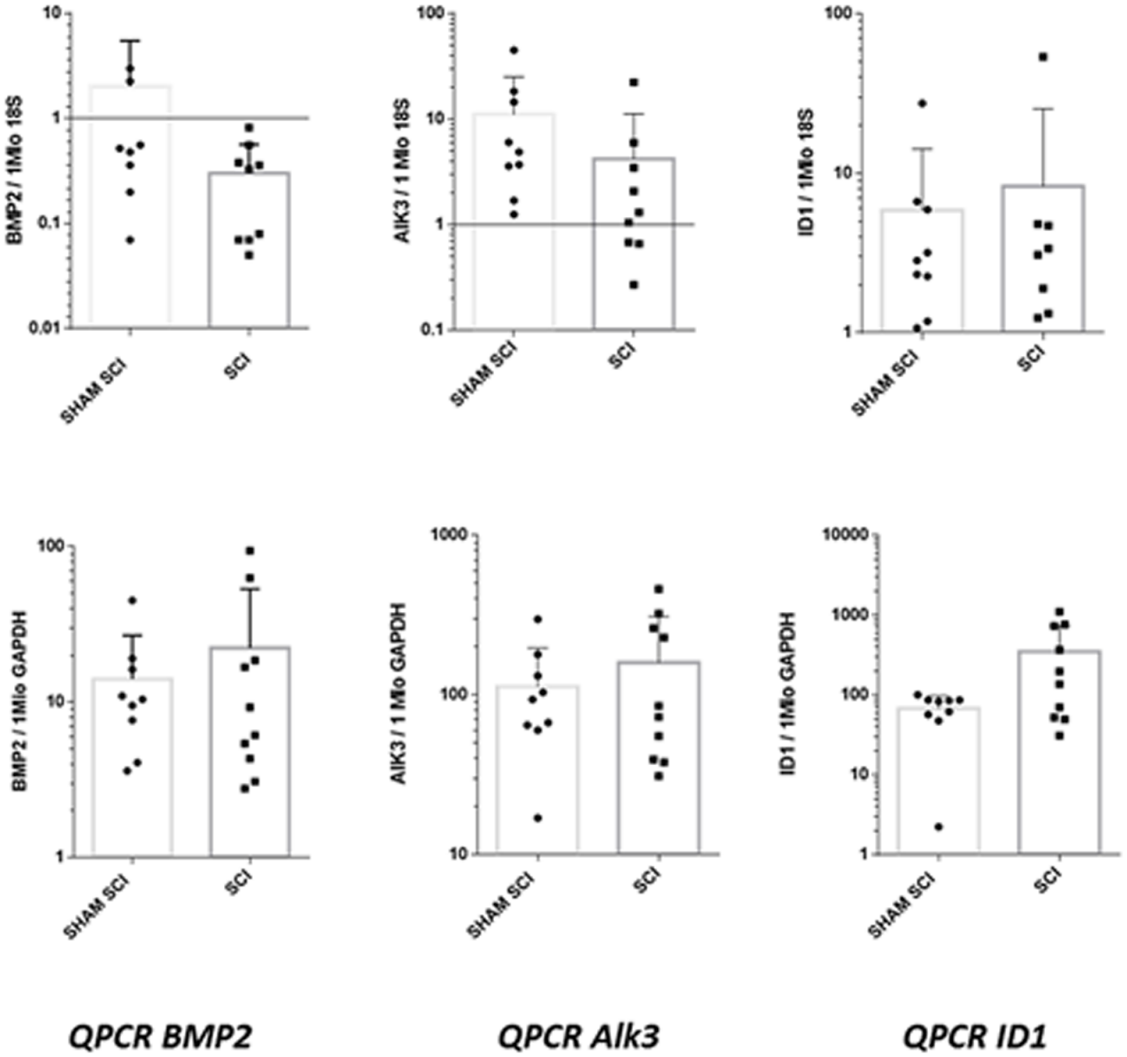
QPCR analyses of BMP signaling pathway in muscular samples of C57bl6 mice, 18 hours after spinal cord injury (SCI) or sham surgery (sham SCI). Studied genes were BMP2, Alk3 (encoding BMP type 1 receptor) and ID1 (encoding inhibitor Of DNA Binding 1, HLH Protein). For each gene, presented results are the means of qPCR triplicate results, reported to housekeeping genes 18S and GADPH. p values were 0.08 and 0.76 respectively for BMP2/18S and BMP2/GADPH; 0.05 and 0.9 respectively for AlK3/18S and AlK3/GADPH; 0.8 and 0.15 respectively for ID1/18S and ID1/GADPH.

Regarding the expression of BMP2, Alk3 and ID1 in muscular samples from SCI and sham SCI mice (Fig 5), qPCR analyses showed no statistically significant differences directly related to spinal cord injury in harvested muscles (S2 Table).

## Discussion

The major goal of this study was to explore the role of peripheral nervous system in an acquired HO mouse model. We successfully developed a right hind limb peripheral denervation procedure. Our results demonstrated that peripheral denervation led to increased heterotopic bone volumes in wild C57Bl6 mice after spinal cord injury and intramuscular CTX injections.

Regarding our experimental design, several points can be discussed. First, significant inter individual variability in HO volume measurements have been previously highlighted in this animal model [22]. Several extrinsic factors can be pointed out, such as non-controlled extra muscular dissemination of CTX after percutaneous injection, but genetic or metabolic components may also be involved. In order to obtain the most robust evaluation of ectopic bone volumes, we tried to minimize bias related to inter individual variability by analyzing paired BV in each mouse. We also realized CTX injections under eye control to prevent any extra muscular leak. Secondly, in order to obtain a complete peripheral hind limb denervation, we decided to realize nerve transection on both femoral and sciatic nerves. We deemed important to suppress axonal transportation towards muscles of both anterior and posterior muscular compartments. We established the efficiency and reproducibility of our peripheral denervation in non-paraplegic control animals (group3), since all of them had a complete motor deficit of denervated paw during the post-operative period and presented with consistent global muscular amyotrophy at day 28.

Finally, group 2 was constituted in order to take into account delayed effects of peripheral denervation on local inflammatory reaction as notified by Stangenberg et al [34]. This author recently demonstrated that nerve transection could alter the transcriptome of joint microvasculature and inhibit inflammation-enhancing vascular leak in K/BxN serum-transferred mice.

Regarding the role of neurologic damage in our mouse model, we first hypothesized that PNS could support neuropeptides transportation from injured spinal cord to muscles. This idea was based on recent experimental data. First, central nerve damage appears to induce systemic release of substance P or calcitonin-gene-related peptide (CGRP) [35, 36] which triggers pro-inflammatory mechanisms and neuronal regeneration [37, 38]. Moreover substance P can be distally released from sensory nerves expressing the transient receptor potential cation channel subfamily V member 1 (TrpV1), also known as the capsaicin receptor, leading to the recruitment of activated platelets, mast cells and neutrophils, which is one inductive step necessary to injury acquired HOs [25–27,39]. Consequently, we rather expected a decrease in HO volumes after peripheral nerves excision, since we thought that interrupting neurotransmitters transportation along peripheral nerves would prevent their intra muscular release. Yet, we observed significantly increased volumes in denervated muscles compared to control paw. We also observed HO formation (n = 3) in group 3, after peripheral denervation and muscular lesions without central nerve damage. These results seem to underline the key role of neuron cell injury, in triggering the release of osteoinductive factors. Rather than having some direct consequences on neuropeptide transport, peripheral nerve section appears to reinforce pathological propensity for intra muscular progenitors to differentiate into bone cells. This effect could be mediated by specific cross-talk between the nervous and immune systems [40–44]. To date, published evidence suggests that injury to the spinal cord elicits a robust intra spinal inflammatory response with major impact on the entire immune system [45–46]. As reviewed by Schwab et al. SCI rapidly induces immune depression syndrome, through the loss of vegetative input to lymphoid and endocrine organs. This down-regulation affects cells of both the innate and adaptive immune systems [47]. Yet macrophages and mast cells have been identified as important regulators in regenerative mechanisms induced by tissue injury [48, 49]. Time-specific infiltration of macrophages during muscle regeneration appears to actively coordinate muscle fiber repair and re-attachment of motoneuron terminals onto damaged fibers through spatiotemporal up-regulation of neural chemo-repellent molecules [50]. These data support the idea that immune cells reprogramming after SCI could influence cellular differentiation of recruited progenitors in damaged muscles, leading to ectopic osteogenesis.

Regarding molecular signals involved in our model, we failed to demonstrate increased expression of BMP signaling pathway in muscles after SCI as described by Kang et al [51]. However experimental designs were focused on different time points, respectively eighteen hours after SCI in our protocol versus 3 days in Kang’s study. This raises the problem of determining the exact time-course of molecular events triggered by SCI. As we were unable to continuously monitor intra muscular changes of BMP expression, we arbitrarily chose to explore an early stage after injury. But further analysis need to be conducted in order to establish robust conclusion on this point. Moreover, considering recent studies on sciatic nerve crush injury models, it seems that BMP signaling pathway contributes to the axonal regeneration of sensory neurons [52–54]. We can easily conceive that in our NHO mouse model, sciatic resection reinforces BMP expression as well as neurogenic inflammation leading to heterotopic ossification.

Our study has several limitations. As we performed global nerve transection we failed to associate increased HO formation with the suppression of a single nerve quality (sympathetic, parasympathetic, or sensory nerves). One might expect that these systems are both involved in HO formation following SCI, but their respective effects remain to be clarified in the particular case of our mouse model. Moreover, preliminary results of molecular analyses need to be completed in order to demonstrate up-regulation of BMP signaling pathway in this NHO model. Further works should aim at identifying cellular sources of BMP and clarifying its regulatory contribution to the cascade of events leading to ectopic osteogenesis.

In conclusion, our study demonstrates that peripheral injury-induced neuro-inflammation leads to increased heterotopic bone volume in a NHO mouse model. This finding supports the idea that propensity to develop ectopic osteogenesis in brain or medullary injured patients could involve disturbed regenerative molecular signals. It remains though a complex multicellular process with several unknown interactions. We succeeded in developing a promising experimental design using neurogenic HO mouse model. This protocol should help us to clarify the NHO pathophysiology and to target preventive therapies.

## Supporting information

S1 Table Micro-CT measurements of bone volumes and statistical analysis. (PDF)

S2 Table Gene expression ratio in real time QPCR and statistical analysis. (PDF)

## References

[1] Aubut J-AL, Mehta S, Cullen N, Teasell RW, ERABI Group, Scire Research Team. A comparison of heterotopic ossification treatment within the traumatic brain and spinal cord injured population: An evidence based systematic review. NeuroRehabilitation. 2011;28(2):151–60. doi: 10.3233/NRE-2011-0643 21447915 10.3233/NRE-2011-0643 PMC3206088

[2] Haran M, Bhuta T, Lee B. Pharmacological interventions for treating acute heterotopic ossification. Cochrane Database Syst Rev. 2004; (4):CD003321 doi: 10.1002/14651858.CD003321.pub3 15495048 10.1002/14651858.CD003321.pub3

[3] Shehab D, Elgazzar AH, Collier BD. Heterotopic ossification. J Nucl Med. Mars 2002;43(3):346–53. 11884494

[4] Stover SL, Niemann KM, Tulloss JR. Experience with surgical resection of heterotopic bone in spinal cord injury patients. Clin Orthop Relat Res. Févr 1991; (263):71–7. 1899639

[5] Genêt F, Jourdan C, Schnitzler A, Lautridou C, Guillemot D, Judet T, et al Troublesome heterotopic ossification after central nervous system damage: a survey of 570 surgeries. PLoS ONE. 2011;6(1):e16632 doi: 10.1371/journal.pone.0016632 21304993 10.1371/journal.pone.0016632 PMC3031592

[6] Salga M, Jourdan C, Durand M-C, Hangard C, Denormandie P, Carlier R-Y, et al Sciatic nerve compression by neurogenic heterotopic ossification: use of CT to determine surgical indications. Skeletal Radiol. Févr 2015;44(2):233–40. doi: 10.1007/s00256-014-2003-6 25218150 10.1007/s00256-014-2003-6

[7] Ranganathan K, Loder S, Agarwal S, Wong VC, Forsberg J, Davis TA, et al Heterotopic Ossification: Basic-Science Principles and Clinical Correlates. J Bone Joint Surg Am. 1 Juill 2015;97(13):1101–11. doi: 10.2106/JBJS.N.01056 26135077 10.2106/JBJS.N.01056 PMC6948799

[8] Sakellariou VI, Grigoriou E, Mavrogenis AF, Soucacos PN, Papagelopoulos PJ. Heterotopic ossification following traumatic brain injury and spinal cord injury: insight into the etiology and pathophysiology. J Musculoskelet Neuronal Interact. Déc 2012;12(4):230–40. 23196266

[9] Genêt F, Ruet A, Almangour W, Gatin L, Denormandie P, Schnitzler A. Beliefs relating to recurrence of heterotopic ossification following excision in patients with spinal cord injury: a review. Spinal Cord. mai 2015;53(5):340–4. 10.1038/sc.2015.20 25687517

[10] Genet F, Marmorat J-L, Lautridou C, Schnitzler A, Mailhan L, Denormandie P. Impact of late surgical intervention on heterotopic ossification of the hip after traumatic neurological injury. J Bone Joint Surg Br. 11 2009;91(11):1493–8. doi: 10.1302/0301-620X.91B11.22305 19880896 10.1302/0301-620X.91B11.22305

[11] Genêt F, Chehensse C, Jourdan C, Lautridou C, Denormandie P, Schnitzler A. Impact of the operative delay and the degree of neurologic sequelae on recurrence of excised heterotopic ossification in patients with traumatic brain injury. J Head Trauma Rehabil. Déc 2012;27(6):443–8. doi: 10.1097/HTR.0b013e31822b54ba 22495100 10.1097/HTR.0b013e31822b54ba

[12] Genêt F, Minooee K, Jourdan C, Ruet A, Denormandie P, Schnitzler A. Troublesome heterotopic ossification and stroke: Features and risk factors. A case control study. Brain Inj. 2015; 29(7–8):866–71. doi: 10.3109/02699052.2015.1005133 25915823 10.3109/02699052.2015.1005133

[13] Anthonissen J, Ossendorf C, Ritz U, Hofmann A, Rommens PM. Animal models for acquired heterotopic ossification. Acta Orthop Belg. Mars 2014;80(1):2–10. 24873078

[14] Kan L, Kessler JA. Animal models of typical heterotopic ossification. J Biomed Biotechnol. 2011;2011:309287 doi: 10.1155/2011/309287 20981294 10.1155/2011/309287 PMC2963134

[15] Urist MR. Bone: formation by autoinduction. Science. 12 11 1965;150(3698):893–9. 5319761 10.1126/science.150.3698.893

[16] Wang RN, Green J, Wang Z, Deng Y, Qiao M, Peabody M, et al Bone Morphogenetic Protein (BMP) signaling in development and human diseases. Genes Dis. 9 2014;1(1):87–105. doi: 10.1016/j.gendis.2014.07.005 25401122 10.1016/j.gendis.2014.07.005 PMC4232216

[17] Glaser DL, Economides AN, Wang L, Liu X, Kimble RD, Fandl JP, et al In vivo somatic cell gene transfer of an engineered Noggin mutein prevents BMP4-induced heterotopic ossification. J Bone Joint Surg Am. Déc 2003;85-A(12):2332–42. 14668502 10.2106/00004623-200312000-00010

[18] Hannallah D, Peng H, Young B, Usas A, Gearhart B, Huard J. Retroviral delivery of Noggin inhibits the formation of heterotopic ossification induced by BMP-4, demineralized bone matrix, and trauma in an animal model. J Bone Joint Surg Am. Janv 2004;86-A(1):80–91. 14711949 10.2106/00004623-200401000-00013

[19] Hayashi C, Hasegawa U, Saita Y, Hemmi H, Hayata T, Nakashima K, et al Osteoblastic bone formation is induced by using nanogel-crosslinking hydrogel as novel scaffold for bone growth factor. J Cell Physiol. Juill 2009;220(1):1–7. doi: 10.1002/jcp.21760 19301257 10.1002/jcp.21760

[20] Gonda K, Nakaoka T, Yoshimura K, Otawara-Hamamoto Y, Harrii K. Heterotopic ossification of degenerating rat skeletal muscle induced by adenovirus-mediated transfer of bone morphogenetic protein-2 gene. J Bone Miner Res. Juin 2000;15(6):1056–65. doi: 10.1359/jbmr.2000.15.6.1056 10841174 10.1359/jbmr.2000.15.6.1056

[21] Peng H, Chen ST, Wergedal JE, Polo JM, Yee JK, Lau KH, et al Development of an MFG-based retroviral vector system for secretion of high levels of functionally active human BMP4. Mol Ther. Août 2001;4(2):95–104. doi: 10.1006/mthe.2001.0423 11482980 10.1006/mthe.2001.0423

[22] Genêt F, Kulina I, Vaquette C, Torossian F, Millard S, Pettit AR, et al Neurological heterotopic ossification following spinal cord injury is triggered by macrophage-mediated inflammation in muscle. J Pathol. Juin 2015;236(2):229–40. doi: 10.1002/path.4519 25712044 10.1002/path.4519

[23] Gautschi OP, Toffoli AM, Joesbury KA, Skirving AP, Filgueira L, Zellweger R. Osteoinductive effect of cerebrospinal fluid from brain-injured patients. J Neurotrauma. Janv 2007;24(1):154–62. doi: 10.1089/neu.2006.0166 17263679 10.1089/neu.2006.0166

[24] Cadosch D, Thyer M, Gautschi OP, Lochnit G, Frey SP, Zellweger R, et al Functional and proteomic analysis of serum and cerebrospinal fluid derived from patients with traumatic brain injury: a pilot study. ANZ J Surg. Août 2010;80(7–8):542–7. doi: 10.1111/j.1445-2197.2010.05268.x 20795970 10.1111/j.1445-2197.2010.05268.x

[25] Kan L, Lounev VY, Pignolo RJ, Duan L, Liu Y, Stock SR, et al Substance P signaling mediates BMP-dependent heterotopic ossification. J Cell Biochem. 10 2011;112(10):2759–72. doi: 10.1002/jcb.23259 21748788 10.1002/jcb.23259 PMC3508732

[26] Ma WH, Liu YJ, Wang W, Zhang YZ. Neuropeptide Y, substance P, and human bone morphogenetic protein 2 stimulate human osteoblast osteogenic activity by enhancing gap junction intercellular communication. Braz J Med Biol Res. Avr 2015;48(4):299–307. doi: 10.1590/1414-431X20144226 25714881 10.1590/1414-431X20144226 PMC4418359

[27] Salisbury E, Rodenberg E, Sonnet C, Hipp J, Gannon FH, Vadakkan TJ, et al Sensory nerve induced inflammation contributes to heterotopic ossification. J Cell Biochem. 10 2011;112(10):2748–58. doi: 10.1002/jcb.23225 21678472 10.1002/jcb.23225 PMC3329372

[28] Li X, Cao X. BMP signaling and skeletogenesis. Ann N Y Acad Sci. Avr 2006;1068:26–40. doi: 10.1196/annals.1346.006 16831903 10.1196/annals.1346.006

[29] Yu PB, Deng DY, Lai CS, Hong CC, Cuny GD, Bouxsein ML, et al BMP type I receptor inhibition reduces heterotopic [corrected] ossification. Nat Med. Déc 2008;14(12):1363–9. doi: 10.1038/nm.1888 19029982 10.1038/nm.1888 PMC2846458

[30] Kan L, Hu M, Gomes WA, Kessler JA. Transgenic mice overexpressing BMP4 develop a fibrodysplasia ossificans progressiva (FOP)-like phenotype. Am J Pathol. 10 2004;165(4):1107–15. doi: 10.1016/S0002-9440(10)63372-X 15466378 10.1016/S0002-9440(10)63372-X PMC1618644

[31] Fukuda T, Kohda M, Kanomata K, Nojima J, Nakamura A, Kamizono J, et al Constitutively Activated ALK2 and Increased SMAD1/5 Cooperatively Induce Bone Morphogenetic Protein Signaling in Fibrodysplasia Ossificans Progressiva. J Biol Chem. 13 Mars 2009;284(11):7149–56. doi: 10.1074/jbc.M801681200 18684712 10.1074/jbc.M801681200 PMC2652274

[32] Hino K, Ikeya M, Horigome K, Matsumoto Y, Ebise H, Nishio M, et al Neofunction of ACVR1 in fibrodysplasia ossificans progressiva. Proc Natl Acad Sci U S A. 15 Déc 2015;112(50):15438–43. doi: 10.1073/pnas.1510540112 26621707 10.1073/pnas.1510540112 PMC4687587

[33] Shore EM, Kaplan FS. Insights from a Rare Genetic Disorder of Extra-Skeletal Bone Formation, Fibrodysplasia Ossificans Progressiva (FOP). Bone. 9 2008;43(3):427–33. doi: 10.1016/j.bone.2008.05.013 18590993 10.1016/j.bone.2008.05.013 PMC2601573

[34] Stangenberg L, Burzyn D, Binstadt BA, Weissleder R, Mahmood U, Benoist C, et al Denervation protects limbs from inflammatory arthritis via an impact on the microvasculature. Proc Natl Acad Sci USA. 5 Août 2014;111(31):11419–24. doi: 10.1073/pnas.1410854111 25049388 10.1073/pnas.1410854111 PMC4128122

[35] Song Y, Bi L, Zhang Z, Huang Z, Hou W, Lu X, et al Increased levels of calcitonin gene-related peptide in serum accelerate fracture healing following traumatic brain injury. Mol Med Rep. Févr 2012;5(2):432–8. doi: 10.3892/mmr.2011.645 22038198 10.3892/mmr.2011.645

[36] Leonard AV, Manavis J, Blumbergs PC, Vink R. Changes in substance P and NK1 receptor immunohistochemistry following human spinal cord injury. Spinal Cord. Janv 2014;52(1):17–23. doi: 10.1038/sc.2013.136 24216617 10.1038/sc.2013.136

[37] Geppetti P, Bertrand C, Ricciardolo FL, Nadel JA, Ricciardolo FM. New aspects on the role of kinins in neurogenic inflammation. Can J Physiol Pharmacol. Juill 1995;73(7):843–7. 8846419 10.1139/y95-115

[38] Kim K-T, Kim H-J, Cho D-C, Bae J-S, Park S-W. Substance P stimulates proliferation of spinal neural stem cells in spinal cord injury via the mitogen-activated protein kinase signaling pathway. Spine J. 1 9 2015;15(9):2055–65. doi: 10.1016/j.spinee.2015.04.032 25921821 10.1016/j.spinee.2015.04.032

[39] Nazarian A, Gu G, Gracias NG, Wilkinson K, Hua XY, Vasko MR, et al Spinal N-methyl-D-aspartate receptors and nociception-evoked release of primary afferent substance P. Neuroscience. 3 Mars 2008;152(1):119–27. doi: 10.1016/j.neuroscience.2007.11.037 18222611 10.1016/j.neuroscience.2007.11.037 PMC2730522

[40] Stoll G, Jander S, Myers RR. Degeneration and regeneration of the peripheral nervous system: from Augustus Waller’s observations to neuroinflammation. J Peripher Nerv Syst. Mars 2002;7(1):13–27. 11939348 10.1046/j.1529-8027.2002.02002.x

[41] Gaudet AD, Popovich PG, Ramer MS. Wallerian degeneration: gaining perspective on inflammatory events after peripheral nerve injury. J Neuroinflammation. 30 Août 2011;8:110 doi: 10.1186/1742-2094-8-110 21878126 10.1186/1742-2094-8-110 PMC3180276

[42] Avellino AM, Hart D, Dailey AT, MacKinnon M, Ellegala D, Kliot M. Differential macrophage responses in the peripheral and central nervous system during wallerian degeneration of axons. Exp Neurol. Déc 1995;136(2):183–98. doi: 10.1006/exnr.1995.1095 7498408 10.1006/exnr.1995.1095

[43] Qin J, Zha G-B, Yu J, Zhang H-H, Yi S. Differential temporal expression of matrix metalloproteinases following sciatic nerve crush. Neural Regen Res. Juill 2016;11(7):1165–71. doi: 10.4103/1673-5374.187059 27630704 10.4103/1673-5374.187059 PMC4994463

[44] Gong L, Wu J, Zhou S, Wang Y, Qin J, Yu B, et al Global analysis of transcriptome in dorsal root ganglia following peripheral nerve injury in rats. Biochem Biophys Res Commun. 9 9 2016;478(1):206–12. doi: 10.1016/j.bbrc.2016.07.067 27450809 10.1016/j.bbrc.2016.07.067

[45] David S, Zarruk JG, Ghasemlou N. Inflammatory pathways in spinal cord injury. Int Rev Neurobiol. 2012;106:127–52. doi: 10.1016/B978-0-12-407178-0.00006-5 23211462 10.1016/B978-0-12-407178-0.00006-5

[46] Beck KD, Nguyen HX, Galvan MD, Salazar DL, Woodruff TM, Anderson AJ. Quantitative analysis of cellular inflammation after traumatic spinal cord injury: evidence for a multiphasic inflammatory response in the acute to chronic environment. Brain. Févr 2010;133(Pt 2):433–47. doi: 10.1093/brain/awp322 20085927 10.1093/brain/awp322 PMC2858013

[47] Schwab JM, Zhang Y, Kopp MA, Brommer B, Popovich PG. The paradox of chronic neuroinflammation, systemic immune suppression and autoimmunity after traumatic chronic spinal cord injury. Exp Neurol. Août 2014;0:121–9. 10.1016/j.expneurol.2014.04.023 PMC4099970 25017893

[48] Cattin A-L, Burden JJ, Van Emmenis L, Mackenzie FE, Hoving JJA, Garcia Calavia N, et al Macrophage-Induced Blood Vessels Guide Schwann Cell-Mediated Regeneration of Peripheral Nerves. Cell. 27 Août 2015;162(5):1127–39. doi: 10.1016/j.cell.2015.07.021 26279190 10.1016/j.cell.2015.07.021 PMC4553238

[49] Shono J, Sakaguchi S, Suzuki T, Do M-KQ, Mizunoya W, Nakamura M, et al Preliminary time-course study of antiinflammatory macrophage infiltration in crush-injured skeletal muscle. Anim Sci J. 11 2013;84(11):744–50. doi: 10.1111/asj.12105 23980916 10.1111/asj.12105

[50] Sakaguchi S, Shono J, Suzuki T, Sawano S, Anderson JE, Do M-KQ, et al Implication of anti-inflammatory macrophages in regenerative moto-neuritogenesis: promotion of myoblast migration and neural chemorepellent semaphorin 3A expression in injured muscle. Int J Biochem Cell Biol. 9 2014;54:272–85. doi: 10.1016/j.biocel.2014.05.032 24886696 10.1016/j.biocel.2014.05.032

[51] Kang H, Dang ABC, Joshi SK, Halloran B, Nissenson R, Zhang X, et al Novel mouse model of spinal cord injury-induced heterotopic ossification. J Rehabil Res Dev. 2014;51(7):1109–18. doi: 10.1682/JRRD.2014.01.0019 25436890 10.1682/JRRD.2014.01.0019

[52] Han B, Zhao J-Y, Wang W-T, Li Z-W, He A-P, Song X-Y. Cdc42 Promotes Schwann Cell Proliferation and Migration Through Wnt/β-Catenin and p38 MAPK Signaling Pathway After Sciatic Nerve Injury. Neurochem Res. 17 Janv 2017; 10.1007/s11064-017-2175-2 28097464

[53] Liu P, Jiang B, Ma J, Lin P, Zhang Y, Shao C, et al S113R mutation in SLC33A1 leads to neurodegeneration and augmented BMP signaling in a mouse model. Dis Model Mech. 1 Janv 2017;10(1):53–62. doi: 10.1242/dmm.026880 27935820 10.1242/dmm.026880 PMC5278525

[54] Ma CHE, Brenner GJ, Omura T, Samad OA, Costigan M, Inquimbert P, et al The BMP coreceptor RGMb promotes while the endogenous BMP antagonist noggin reduces neurite outgrowth and peripheral nerve regeneration by modulating BMP signaling. J Neurosci. 14 Déc 2011;31(50):18391–400. doi: 10.1523/JNEUROSCI.4550-11.2011 22171041 10.1523/JNEUROSCI.4550-11.2011 PMC3243947

