# Supplementary material for: Peripheral denervation participates in heterotopic ossification in a spinal cord injury model
Source: PLoS One. 2017 Aug 30;12(8):e0182454. doi: 10.1371/journal.pone.0182454 (PMC5576715; doi:10.1371/journal.pone.0182454)
Supplement: S1 Table — (PDF) [file pone.0182454.s001.pdf]

**Bone volumes (microCT, mm3)**	data group 1		data group 2		data group 3	
sample	RHL	LHL	RHL	LHL	RHL	LHL
1	**1,26**	**0,38**	0	0	**1,34**	**0,72**
2	**2,1**	**1,21**	0	0	**0,65**	**0,2**
3	**0,77**	**0,17**	0	0	**0,25**	**0,09**
4	**2,25**	**0,86**	0	0	**0**	**0**
5	**1,37**	**0,22**	0	0	**0**	**0**
6	**1,11**	**0,52**	0	0	**0**	**0**
Number of values	6	6			3	3
Minimum	0,77	0,17			0,25	0,09
25% Percentile	1,025	0,2075			0,25	0,09
Median	1,315	0,45			0,65	0,2
75% Percentile	2,138	0,9475			1,34	0,72
Maximum	2,25	1,21			1,34	0,72
Mean	**1,477**	**0,56**			**0,7467**	**0,3367**
Std. Deviation	**0,5794**	**0,4035**			**0,5514**	**0,3365**
Std. Error of Mean	0,2366	0,1647			0,3183	0,1943
Lower 95% CI	0,8686	0,1365			-0,6231	-0,4992
Upper 95% CI	2,085	0,9835			2,116	1,173

	data group 1	data group 3
Wilcoxon matched-pairs signed rank test		
P value	**0,0313**	0,25
Exact or approximate P value?	Exact	Exact
P value summary	*	ns
Significantly different? (P < 0.05)	Yes	No
One- or two-tailed P value?	Two-tailed	Two-tailed
Sum of positive, negative ranks	0,0 , -21,00	0,0 , -6,000
Sum of signed ranks (W)	-21	-6
Median of differences		
Median	-0,885	-0,45
How effective was the pairing?		
rs (Spearman)	0,7143	1
P value (one tailed)	0,0681	0,1667
P value summary	ns	ns
Significant correlation? (P > 0.05)	Yes	Yes
