# Supplementary material for: Peripheral denervation participates in heterotopic ossification in a spinal cord injury model
Source: PLoS One. 2017 Aug 30;12(8):e0182454. doi: 10.1371/journal.pone.0182454 (PMC5576715; doi:10.1371/journal.pone.0182454)
Supplement: S2 Table — (PDF) [file pone.0182454.s002.pdf]

**Gene expression ratio (QPCR)**	BMP2 18S		BMP2 GAPDH		Alk3 18S		Alk3 GAPDH		ID1 18S		ID1 GAPDH	
sample	SHAM SCI	SCI	SHAM SCI	SCI	SHAM SCI	SCI	SHAM SCI	SCI	SHAM SCI	SCI	SHAM SCI	SCI
1	0,56	0,08	45,18475	9,267337	3,71	0,66	298,432	72,86484	1,07	1,24	86,28071	136,9199
2	10,84	0,82	4,088694	16,78911	45,05	22,33	16,99067	459,7387	5,92	53,75	2,231485	1106,743
3	2,26	0,05	16,23829	18,54039	14,51	0,68	104,0983	262,2426	6,65	1,9	47,72474	729,4543
4	0,36	0,33	9,500276	3,083675	3,58	6	93,7391	55,2407	3,18	3,37	83,19928	30,99007
5	0,07	0,07	3,613863	2,783436	1,25	1,05	60,29492	39,55104	1,18	1,32	56,9346	49,5203
6	3	0,38	10,95613	94,08148	18,37	1,31	67,08678	323,9243	27,51	3,08	100,4533	763,8598
7	0,48	0,36	10,42677	4,333978	6,04	2,08	131,8018	85,2944	2,84	4,7	61,96968	197,4792
8	0,52	0,56	19,10367	5,415516	4,89	3,47	179,6312	31,02634	2,32	4,8	85,04094	70,20524
9	0,2	0,07	7,661071	6,121233	1,7	0,27	64,82316	37,91399	2,26	0,43	86,34801	52,39146
10				62,8377				228,5956				367,5162
Number of values	9	9	9	9	9	9	9	9	9	9	9	9
Minimum	0,07	0,05	3,614	2,783	1,25	0,27	16,99	31,03	1,07	0,43	2,231	30,99
25% Percentile	0,28	0,07	5,875	4,021	2,64	0,67	62,56	39,14	1,72	1,28	52,33	51,67
Median	0,52	0,33	10,43	7,694	4,89	1,31	93,74	79,08	2,84	3,08	83,2	167,2
75% Percentile	2,63	0,47	17,67	29,61	16,44	4,735	155,7	277,7	6,285	4,75	86,31	738,1
Maximum	10,84	0,82	45,18	94,08	45,05	22,33	298,4	459,7	27,51	53,75	100,5	1107
Mean	2,032	0,3022	14,09	22,33	11,01	4,206	113	159,6	5,881	8,288	67,8	350,5
Std. Deviation	3,454	0,2659	12,71	30,97	14,04	7,032	83,71	149,8	8,337	17,12	29,88	382,4
Std. Error of Mean	1,151	0,08863	4,236	9,792	4,681	2,344	27,9	47,38	2,779	5,706	9,959	120,9
Lower 95% CI of mean	-0,6226	0,09785	4,317	0,1735	0,2165	-1,2	48,65	52,45	-0,5269	-4,869	44,83	76,98
Upper 95% CI of mean	4,687	0,5066	23,85	44,48	21,81	9,611	177,3	266,8	12,29	21,45	90,76	624
P value	0,0881		0,7655		0,0502		0,9438		0,8427		0,1545	
Exact or approximate P value?	Exact		Exact		Exact		Exact		Exact		Exact	
P value summary	ns		ns		ns		ns		ns		ns	
Significantly different? (P < 0.05)	No		No		No		No		No		No	
One- or two-tailed P value?	Two-tailed		Two-tailed		Two-tailed		Two-tailed		Two-tailed		Two-tailed	
Sum of ranks in column A,B	105,0 , 66,00		94,00 , 96,00		108,0 , 63,00		89,00 , 101,0		88,00 , 83,00		72,00 , 118,0	
Mann-Whitney U	21		41		18		44		38		27	
